# A Stochastic Total Least Squares Solution of Adaptive Filtering Problem

**DOI:** 10.1155/2014/625280

**Published:** 2014-02-03

**Authors:** Shazia Javed, Noor Atinah Ahmad

**Affiliations:** ^1^School of Mathematical Science, Universiti Sains Malaysia, 11800 Penang, Malaysia; ^2^Lahore College for Women University, Lahore 54000, Pakistan

## Abstract

An efficient and computationally linear algorithm is derived for total least squares solution of adaptive filtering problem, when both input and output signals are contaminated by noise. The proposed total least mean squares (TLMS) algorithm is designed by recursively computing an optimal solution of adaptive TLS problem by minimizing instantaneous value of weighted cost function. Convergence analysis of the algorithm is given to show the global convergence of the proposed algorithm, provided that the stepsize parameter is appropriately chosen. The TLMS algorithm is computationally simpler than the other TLS algorithms and demonstrates a better performance as compared with the least mean square (LMS) and normalized least mean square (NLMS) algorithms. It provides minimum mean square deviation by exhibiting better convergence in misalignment for unknown system identification under noisy inputs.

## 1. Introduction

Ordinary least squares methods are extensively used in many signal processing applications to extract the system parameters from input/output data [1, 2]. These methods yield an unbiased solution of adaptive least squares problem having no interference in both inputs and outputs or having interference only in the outputs of the unknown system and clean inputs. However, if interference exists in both input and output of the unknown system or adaptive filtering problem, the ordinary least squares solution gets biased [3].

Total least squares (TLS) method [4] is an efficient technique to achieve an unbiased estimate of the system parameters when both input and output are contaminated by noise. Golub and Van Loan [5] provided an analytical procedure to get an unbiased solution of the TLS problem using singular value decomposition (SVD) of data matrices. This technique is extensively used in data processing and control applications [4, 6, 7]. However, application of TLS methods in signal processing is still limited because computation of SVD requires a high complexity of *O*(*N* ^3^) for an *N* × *N* matrix.

TLS solutions of adaptive filtering problem gained importance after the pioneer work done by Pisarenko [8]. He presented an efficient solution of adaptive TLS problem by adaptively computing the eigenvector corresponding to smallest eigenvalue of augmented input/output signal's autocorrelation matrix. Since then, several algorithms have been proposed based on the adaptive implementations of Pisarenko. The adaptive TLS algorithms proposed in [9–11] are able to achieve an unbiased TLS solution of adaptive filtering problem with a complexity of *O*(*N*). However they are sensitive to the correlation properties of input signals and have a drawback of bad performance under correlated inputs.

In this paper, an iterative algorithm is presented to find an optimal TLS solution of adaptive FIR filtering problem. A stochastic technique similar to that of least mean squares (LMS) algorithm of adaptive least squares filtering is employed to develop a total least mean squares (TLMS) algorithm for adaptive total least squares problem. Instead of basing the approach on the minimum mean squares error as the LMS algorithm does, the proposed (TLMS) algorithm is based on the total mean squares, obtained by minimizing the weighted cost function for the TLS solution of adaptive filtering problem. The proposed algorithm has maintained the *O*(*N*) complexity of adaptive TLS algorithms with an additional quality of having steady state convergence under correlated inputs. Convergence analysis is presented to show the global convergence of the proposed algorithm under all kinds of inputs provided the stepsize parameter is suitably chosen.

This paper is outlined as follows: we start with a mathematical formulation of adaptive least squares problem in Section 2 and derivation of the TLMS algorithm is given in Section 3, including its convergence analysis in Section 3.1. After that efficiency of the proposed algorithm is tested in Section 4 by applying it for an unknown system identification problem and comparing the results with conventional LMS and normalized LMS (NLMS) algorithms. Concluding remarks are given in Section 5.

## 2. Mathematical Formulation of Adaptive Total Least Squares Problem

Consider an unknown system to be identified by adaptive FIR filter of length *N* and response vector **w**_*n*_, at time *n*, with an assumption that both input and output are corrupted by an additive white Gaussian noise (AWGN). The noise free input vector **a**_*n*_ ∈ *ℛ* ^*N*^ is formed from the input signals *u*(*n*), such that

(1) $$\begin{array}{c} a_{n}={\left[u\left(n\right),u\left(n-1\right),\ldots ,u\left(n-N+1\right)\right]}^{T}\in {ℛ}^{N}. \end{array}$$

The desired output of the unknown system is then given by

(2) $$\begin{array}{c} \tilde{s}\left(n\right)=s\left(n\right)+\Delta s\left(n\right), \end{array}$$

where *s*(*n*) = **w**_*n*_^*T*^ **a**_*n*_ is system's output and Δ*s*(*n*) an added white Gaussian noise of zero mean and variance *σ*_Δ*s*_^2^.

**Figure 1:**
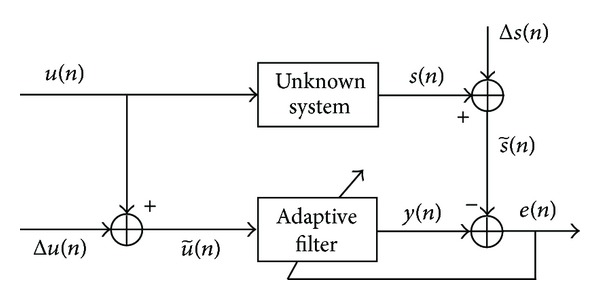
An unknown system identification model for adaptive filtering of noisy input signals.

The primary assumption of an adaptive least squares (ALS) problem is that perturbations occur in the output signals only and that the input signals are exactly known. This assumption is not practical enough, because perturbations due to sampling or modeling or measurement errors affect the input signals too. A sensible choice to overcome such situations is to introduce perturbations in input signals in addition to perturbations of output signals. A schematic diagram of an adaptive filter with perturbed input is depicted in Figure 1.

If Δ**a**_*n*_ = [Δ*u*(*n*), Δ*u*(*n* − 1),…, Δ*u*(*n* − *N* + 1)]^*T*^ ∈ *ℛ* ^*N*^, denote the perturbations in input vector **a**_*n*_, where Δ*u*(*n*) is an additive white Gaussian noise (uncorrelated from the output noise) of zero mean and variance *σ*_Δ*u*_^2^, then noisy input vector is

(3) $$\begin{array}{c} {\tilde{a}}_{n}=a_{n}+\Delta a_{n}. \end{array}$$

It is clear from Figure 1 that for every input signal $\tilde{u}(n)=u(n)+\Delta u(n)$, the filter produces an estimated output $y(n)=w_{n}^{T}{\tilde{a}}_{n}$, which is compared with $\tilde{s}(n)$ to produce a least squares error signal $e(n)=y(n)-\tilde{s}(n)$. Define the autocorrelation matrix $R_{{\tilde{a}}_{n}}$ of noisy input vector ${\tilde{a}}_{n}$ as $R_{{\tilde{a}}_{n}}=E\{{\tilde{a}}_{n}{\tilde{a}}_{n}^{T}\}$ and the cross-correlation vector of output signal with ${\tilde{a}}_{n}$ as $p_{{\tilde{a}}_{n}}=E\{\tilde{s}(n){\tilde{a}}_{n}\}$.

At this stage the least squares solution, obtained by minimizing the cost function *J* = *E*{*e* ^2^(*n*)}, gives a poor estimation of the solution of adaptive filtering problem because of the presence of noise in filter input. Casting adaptive filtering problem as total least squares problem can, however, restructure the poor estimation of solution under noisy input [10, 11]. The following definition is made to adopt a more general signal model for ATLS-based filtering.

**Definition 1 (augmented data vector)**

Define an (*N* + 1) × 1 augmented data vector ${\tilde{z}}_{n}$ as

(4) $$\begin{array}{c} {\tilde{z}}_{n}={\left[{\tilde{a}}_{n}^{T}:\tilde{s}(n)\right]}^{T}=\left(\begin{array}{c} {\tilde{a}}_{n}\\ \tilde{s}\left(n\right) \end{array}\right). \end{array}$$

An alternate form of *e*(*n*), in terms of augmented data vector of Definition 1, is obtained as follows:

(5) e(n)=y(n)−s~(n)=wnTa~n−s~(n)=[wnT−1](a~ns~(n))=w~nTz~n,

where ${\tilde{w}}_{n}={\left[w_{n}^{T}-1\right]}^{T}$ denote the (*N* + 1) × 1 extended parameter vector.

The TLS solution of adaptive filtering problem is an eigenvector associated with the smallest eigenvalue of extended autocorrelation matrix ${\tilde{R}}_{n}$:

(6) $$\begin{array}{c} {\tilde{R}}_{n}=E\left\{{\tilde{z}}_{n}{\tilde{z}}_{n}^{T}\right\}=\left(\begin{array}{cc} R_{{\tilde{a}}_{n}} & p_{{\tilde{a}}_{n}}\\ p_{{\tilde{a}}_{n}}^{T} & \sigma_{\tilde{s}(n)}^{2} \end{array}\right), \end{array}$$

where $\sigma_{\tilde{s}(n)}^{2}=E\{\tilde{s}(n)\tilde{s}(n)\}$.

Instead of minimizing the mean square error *E*{*e* ^2^(*n*)}, adaptive total least squares problem is concerned with minimizing the total mean square error *E*{*η* ^2^(*n*)} and cost function $J({\tilde{w}}_{n})=E\{e^{2}(n)\}$, where the total error *η*(*n*) is given by

(7) $$\begin{array}{c} \eta \left(n\right)=\frac{e\left(n\right)}{\sqrt{{\tilde{w}}_{n}^{T}{\tilde{w}}_{n}}}=\frac{{\tilde{w}}_{n}^{T}{\tilde{z}}_{n}}{\sqrt{{\tilde{w}}_{n}^{T}{\tilde{w}}_{n}}}=\frac{{\tilde{z}}_{n}^{T}{\tilde{w}}_{n}}{\sqrt{{\tilde{w}}_{n}^{T}{\tilde{w}}_{n}}}. \end{array}$$

The TLS cost function $\tilde{J}({\tilde{w}}_{n})$ is then defined in terms of total error as

(8) $$\begin{array}{c} \tilde{J}=E\left\{\eta^{2}\left(n\right)\right\}=E\left\{\frac{{\tilde{w}}_{n}^{T}{\tilde{z}}_{n}{\tilde{z}}_{n}^{T}{\tilde{w}}_{n}}{{\tilde{w}}_{n}^{T}{\tilde{w}}_{n}}\right\}=\frac{{\tilde{w}}_{n}^{T}{\tilde{R}}_{n}{\tilde{w}}_{n}}{{\tilde{w}}_{n}^{T}{\tilde{w}}_{n}}. \end{array}$$

The adaptive total least squares problem is a minimization problem of the form [10, 11]:

(9) $$\begin{array}{c} \underset{{\tilde{w}}_{n}\in {ℛ}^{N+1}}{\min}\tilde{J}=\underset{{\tilde{w}}_{n}\in {ℛ}^{N+1}}{\min}\frac{{\tilde{w}}_{n}^{T}{\tilde{R}}_{n}{\tilde{w}}_{n}}{{\tilde{w}}_{n}^{T}{\tilde{w}}_{n}}. \end{array}$$

Note that an optimal solution **w**_opt_ of the TLS problem (9) is an eigenvector corresponding to the smallest eigenvalue of ${\tilde{R}}_{n}$. In practice SVD technique is used to solve TLS problems since it offers lower sensitivity to the computational errors; however, it is computationally expensive [5]. An alternate choice to estimate eigenvector corresponding to smallest eigenvalue is to use an adaptive algorithm [1, 2].

## 3. Derivation of Total LMS Algorithm for Adaptive Filtering Problem

In adaptive least squares problem, conventional LMS algorithm is a steepest descent method which uses an instantaneous cost function *J* = *e* ^2^(*n*) for computation of gradient vector [1]. Using a similar implementation in TLS problem, the total LMS (TLMS) algorithm is obtained by having an instantaneous estimate of the cost function (8) as $\tilde{J}=\eta^{2}(n)$. The recursive update equation of TLMS algorithm is then given as

(10) $$\begin{array}{c} {\tilde{w}}_{n+1}={\tilde{w}}_{n}-\mu \nabla_{{\tilde{w}}_{n}}\tilde{J}, \end{array}$$

where *μ* is the stepsize parameter or convergence parameter. Note that

(11) ∇w~nJ~=∂∂w~n(η(n)2)=2η(n)∂∂w~nη(n)=2η(n)∂∂w~n(w~nTz~nw~nTw~n)=2η(n)w~nTw~n{z~nw~nTw~n−w~nTz~nw~nw~nTw~n}=2η(n)(w~nTw~n)3/2{z~n·w~nTw~n−w~nTz~n·w~n}.

Using $e(n)={\tilde{z}}_{n}^{T}{\tilde{w}}_{n}={\tilde{w}}_{n}^{T}{\tilde{z}}_{n}$ and $||{\tilde{w}}_{n}||=\sqrt{{\tilde{w}}_{n}^{T}{\tilde{w}}_{n}}$, then above equation becomes

(12) $$\begin{array}{c} \nabla_{{\tilde{w}}_{n}}\tilde{J}=\frac{2}{{||{\tilde{w}}_{n}||}^{4}}\left\{{||{\tilde{w}}_{n}||}^{2}e\left(n\right)\cdot {\tilde{z}}_{n}-e{\left(n\right)}^{2}\cdot {\tilde{w}}_{n}\right\}. \end{array}$$

Substituting (12) in (10), the updated equation of TLMS algorithm becomes

(13) $$\begin{array}{c} {\tilde{w}}_{n+1}={\tilde{w}}_{n}+\frac{2e\left(n\right)\cdot \mu }{{||{\tilde{w}}_{n}||}^{4}}\left\{e\left(n\right)\cdot {\tilde{w}}_{n}-{||{\tilde{w}}_{n}||}^{2}{\tilde{z}}_{n}\right\}. \end{array}$$

Once ${\tilde{w}}_{n+1}$ is computed using (13), the TLS solution update **w**_*n*+1_ is obtained by the following formula:

(14) $$\begin{array}{c} w_{n+1}=-\frac{{\tilde{w}}_{n+1}\left(1:N\right)}{{\tilde{w}}_{n+1}\left(N+1\right)}. \end{array}$$

The detailed TLMS algorithm is summarized in Table 1. A complexity measure of the algorithms shows that it is a computationally linear algorithm, requiring a total of 6*N* + 9 multiplications/divisions per iteration. This computational simplicity of adaptive TLMS algorithm makes it a better choice than computationally expensive SVD based TLS algorithm, which requires 6*N* ^3^ computations per iteration [5].

### 3.1. Convergence Analysis

In (13), inner product with ${\tilde{z}}_{n}$ yields,

(15) w~n+1Tz~n=(w~n+2e(n)·μ||w~n||4{e(n)·w~n−||w~n||2z~n})Tz~n=w~nTz~n+2e(n)·μ||w~n||4{e(n)·w~nTz~n−||w~n||2z~nTz~n}=e(n)+2e(n)·μ||w~n||4{|e(n)|2−||w~n||2||z~n||2}=e(n)+2e(n)·μ||w~n||2{|η(n)|2−||z~n||2}.

Since $e(n)={\tilde{w}}_{n}^{T}{\tilde{z}}_{n}$, Cauchy-Schwarz inequality [12] gives

(16) $$\begin{array}{c} {\left|e\left(n\right)\right|}^{2}\leq {||{\tilde{w}}_{n}||}^{2}{||{\tilde{z}}_{n}||}^{2}; \end{array}$$

that is,

(17) $$\begin{array}{c} {\left|\frac{e\left(n\right)}{||{\tilde{w}}_{n}||}\right|}^{2}\leq {||{\tilde{z}}_{n}||}^{2},\\ {\left|\eta \left(n\right)\right|}^{2}\leq {||{\tilde{z}}_{n}||}^{2},\\ {\left|\eta \left(n\right)\right|}^{2}-{||{\tilde{z}}_{n}||}^{2}\leq 0. \end{array}$$

Let $\delta_{n}={||{\tilde{z}}_{n}||}^{2}-|\eta (n){|}^{2}\geq 0$ then (15) becomes:

(18) w~n+1Tz~n=e(n)+2e(n)·μ||w~n||2{−δn}={1−2μδn||w~n||2}e(n)

or,

(19) $$\begin{array}{c} {\tilde{w}}_{n+1}^{T}{\tilde{z}}_{n}=\left\{1-\frac{2\mu \delta_{n}}{{||{\tilde{w}}_{n}||}^{2}}\right\}{\tilde{w}}_{n}^{T}{\tilde{z}}_{n}. \end{array}$$

Since ${\tilde{z}}_{n}\neq 0$,

(20) $$\begin{array}{c} {\tilde{w}}_{n+1}=\left\{1-\frac{2\mu \delta_{n}}{{||{\tilde{w}}_{n}||}^{2}}\right\}{\tilde{w}}_{n} \end{array}$$

which shows that $\left\{{\tilde{w}}_{n}\right\}$ is a geometric progression. It would converge to an optimal solution if

(21) $$\begin{array}{c} |1-\frac{2\mu \delta_{n}}{{||{\tilde{w}}_{n}||}^{2}}|<1 \end{array}$$

or

(22) $$\begin{array}{c} 0<\frac{\mu }{{||{\tilde{w}}_{n}||}^{2}}<\frac{1}{\delta_{n}}. \end{array}$$

This shows that the proposed algorithm is a variable stepsize algorithm, with $\tilde{\mu }=\mu /{||{\tilde{w}}_{n}||}^{2}$. An appropriate way to choose *μ* is to initialize the algorithm such that ||**w**_*o*_ − **w**_opt_|| is less than 2||**w**_opt_|| [13]. According to this result for **w**_*o*_ = 0, $||{\tilde{w}}_{o}||=1$ and $\tilde{\mu }=\mu$, while $\delta_{o}={||{\tilde{z}}_{n}||}^{2}-\tilde{s}(n)>0$.

## 4. Application of TLMS Algorithm in System Identification

**Figure 2:**
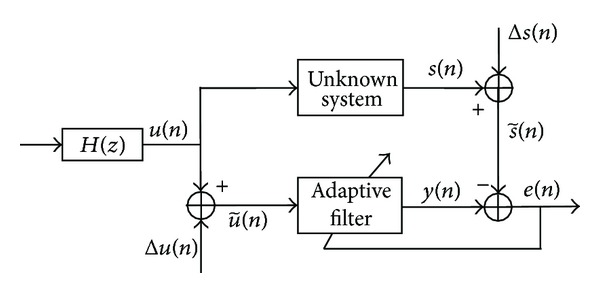
An unknown system identification model for adaptive filtering of correlated noisy signals.

To examine the performance of proposed TLMS algorithm, an unknown system identification model, shown in Figure 2, is used.

A white Gaussian input signal of variance *σ* ^2^ = 1 is passed through a coloring filter with frequency response [1]:

(23) $$\begin{array}{c} H\left(z\right)=\frac{\sqrt{1\;-\;\alpha^{2}}}{1-\alpha z^{-1}}, \end{array}$$

where |*α*| < 1, *α* is a correlation parameter and controls the eigenvalue spread of input signals. *α* = 0 corresponds to the case when eigenvalue spread of input signals is close to 1, and eigenvalue spread increases with an increase in the value of *α*.

A white Gaussian noise of SNR = 30 dB is added in the input signal *u*(*n*) to get noisy signal $\tilde{u}(n)$, and an output signal $\tilde{s}(n)$ is obtained by corrupting the output signal *s*(*n*) with an additive white Gaussian noise of SNR 30 dB. Proposed TLMS algorithm is compared with LMS and NLMS algorithms of [1] to get an FIR vector for a filter of length *N* = 10. Least squares misalignment ||**w**_*n*_ − **w**_opt_|| is compared with the total least squares misalignment $||w_{n}-w_{\text{opt}}||/\sqrt{{\tilde{w}}_{n}^{T}{\tilde{w}}_{n}}$, and simulations results are recorded for 2000 iterations with an ensemble average of 1000 independent runs.

### 4.1. Convergence Behavior Corresponding to Stepsize Parameter *μ*

**Figure 3:**
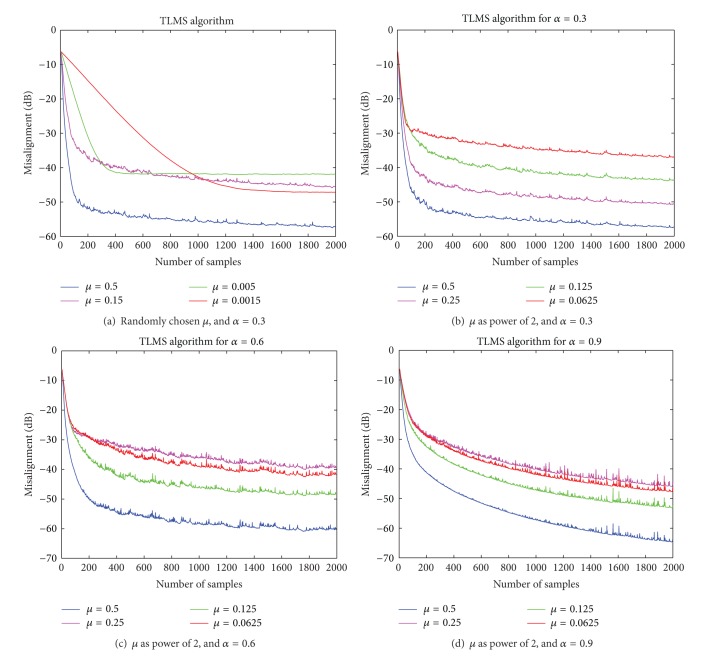
Learning curves of total misalignment of TLMS algorithm.

Although TLMS algorithm converges for all values of *μ*, satisfying (22), but steady state convergence TLMS algorithm is observed when stepsize parameter *μ* is a power of 2. In Figures 3(b)–3(d), four learning curves of total misalignment of TLMS algorithm are shown, corresponding to *μ* = 0.5,0.25,0.125, and 0.0625, and it is observed that robustness increases uniformly with an increase in the value of *μ*. On the other hand if *μ* is chosen randomly, then a change in the convergence behavior is random, though Figure 3(a) shows that algorithm still converges.

### 4.2. Convergence Behavior Corresponding to Correlation Parameter *α*

To check effect of changes in correlation parameter *α* on the steady state convergence behavior of TLMS algorithm, different simulations are presented in Figure 3, each showing four learning curves of total misalignment of TLMS algorithm corresponding to *μ* = 0.5,0.25,0.125, and 0.0625. In the first two simulations *α* = 0.3 in Figures 3(a) and 3(b), it is 0.6 in Figure 3(c), and 0.9 in Figure 3(d). It is clear from the results of all these simulation curves that increase in correlation of data signals has not affected the steady state performance of the algorithm. Although the convergence speed seems to slow down, but all the curves converge to optimal solution.

### 4.3. Comparison

**Figure 4:**
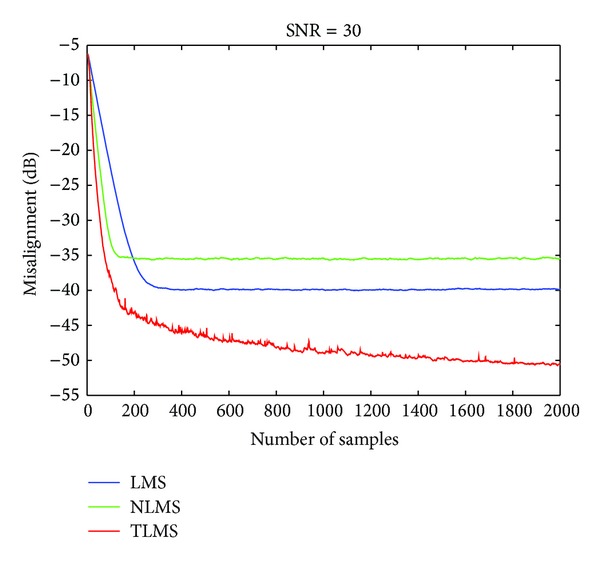
Comparison of TLMS, LMS, and NLMS algorithms.

Figure 4 shows the comparison of misalignment of three algorithms, that is, LMS, NLMS, and TLMS algorithms. The first two compute a least squares solution of adaptive total least squares problem, while the third one computes TLS solution of adaptive total least squares problem. Taking *α* = 0.3, stepsize parameter for LMS algorithm is chosen as 0.015, for NLMS algorithm as 0.3, and for TLMS algorithm, it is 0.25. The results in this simulation show that the convergence of TLMS algorithm increases with an increase in the iteration, and it presents a better solution of adaptive TLS problem.

## 5. Conclusion

In this paper, an efficient TLMS algorithm is presented for the total least squares solution of adaptive filtering problem. The proposed algorithm is derived by using cost function of weighted instantaneous error signals and an efficient computation of misalignment in terms of mean squares deviation. TLMS algorithm has better ability to tackle with perturbations of both input and output signals, because it is chiefly derived for the purpose. Since in real life problems, both input and output signals are contaminated by noise, therefore TLMS algorithm has great applicability. Convergence analysis shows that the proposed algorithm has global convergence, provided that the stepsize parameter is chosen appropriately. Furthermore, it is computationally simple and requires only *O*(*N*) complexity, while other algorithms for TLS problems either require higher complexity or are sensitive to correlation properties of data signals.

## Figures and Tables

**Table 1:** LMS-total (TLMS) algorithm for adaptive filtering.

Algorithm	×/÷	+/−
***Initialization***		
**w**_*o*_ = 0	…	…
${\tilde{w}}_{o}={\left[w_{o}^{T}\;\;\;\;-1\right]}^{T}$	…	…
***Update***		
for *n* = 0,1, 2,…		
${\tilde{z}}_{n}={\left[\;\;{\tilde{a}}_{n}^{T}:\tilde{s}\left(n\right)\;\;\right]}^{T}$	…	…
$e(n)={\tilde{w}}_{n}^{T}{\tilde{z}}_{n}$	*N* + 1	*N*
${\mathrm{norm}}_{\text{sq}}={\tilde{w}}_{n}^{T}{\tilde{w}}_{n}$	*N* + 1	*N*
${\tilde{w}}_{n+1}={\tilde{w}}_{n}+\frac{2\mu \;e\left(n\right)}{{\mathrm{norm}}_{\text{sq}}^{\text{2}}}\left\{e\left(n\right)\cdot {\tilde{w}}_{n}-{\mathrm{norm}}_{\text{sq}}\cdot {\tilde{z}}_{n}\right\}$	3*N* + 7	2*N* + 2
$w_{n+1}=-\frac{{\tilde{w}}_{n+1}(1:N)}{{\tilde{w}}_{n+1}(N+1)}$	*N*	…

Total	6*N* + 9	4*N* + 2
